# The Role of Immunotherapy and Immune Modulators in Hormone-Positive Breast Cancer: Implications for Localized and Metastatic Disease

**DOI:** 10.3390/jcm14124322

**Published:** 2025-06-17

**Authors:** Justin O’Farrell, Caroline Lapp, Heidi Kuznia, Muhammad Z. Afzal

**Affiliations:** 1 Department of Internal Medicine, Dartmouth-Hitchcock Medical Center, Lebanon, NH 03766, USA; caroline.e.lapp@hitchcock.org (C.L.); heidi.m.kuznia@hitchcock.org (H.K.); 2 Section of Hematology/Oncology, Dartmouth-Hitchcock Medical Center, Lebanon, NH 03766, USA; muhammad.z.afzal@hitchcock.org

**Keywords:** hormone-positive breast cancer, immunotherapy, immune checkpoint inhibitors, tumor microenvironment, endocrine resistance, metastatic breast cancer, immune modulators

## Abstract

**Background**: Hormone receptor-positive (HR+) breast cancer, which accounts for approximately 70% of all breast cancer cases, is primarily treated with endocrine therapies as monotherapy or in combination with other targeted therapies and traditional cytotoxic therapy. While these therapies have significantly improved survival outcomes, resistance often develops, especially in metastatic disease where treatment options are limited. Immunotherapy, particularly immune checkpoint inhibitors, has emerged as a promising adjunct to traditional therapies in triple negative breast cancer. However, its role in HR+ breast cancer is not well established yet and is under active investigation. In addition, a deeper understanding of the tumor microenvironment (TME) and its role in immune evasion has spurred interest in immune modulators as additional potential therapeutic strategies for HR+ breast cancers. **Objective**: This review aims to synthesize the current evidence on the role of immunotherapy in HR+ breast cancer, with a focus on its clinical application in both localized and metastatic disease. It also explores the impact of immune modulators within the TME, highlighting their potential to improve the efficacy of immunotherapy in this subtype of breast cancer. **Methods**: A thorough review of recent clinical trials, preclinical studies, and meta-analyses was conducted. Studies published from 2015 to 2024 were included to provide the most up-to-date perspectives. **Key Findings**: While immune checkpoint inhibitors like pembrolizumab and atezolizumab have shown promising results in combination with chemotherapy for triple negative breast cancer, their efficacy in HR+ breast cancer has generally been modest. However, recent studies indicate that such therapies may help overcome endocrine resistance in metastatic disease. Furthermore, immune modulators, including cytokines and myeloid-derived suppressor cell inhibitors, are being investigated for their ability to reshape the TME, potentially enhancing the immune response and improving treatment outcomes. Despite these advances, challenges remain in identifying predictive biomarkers and managing immune-related adverse events. **Conclusions**: Immunotherapy holds potential for improving outcomes in HR+ breast cancer, particularly in metastatic settings where treatment options are limited. The integration of immune modulators to enhance therapeutic efficacy in HR+ breast cancer, along with ongoing research into biomarkers, promises to refine patient selection and improve clinical outcomes.

## 1. Introduction

Hormone receptor-positive (HR+) breast cancer is the most common subtype of breast cancer, comprising approximately 70% of all diagnosed breast cancer cases. Standard therapies, including selective estrogen receptor modulators (e.g., tamoxifen) and aromatase inhibitors, have significantly reduced recurrence rates and improved overall survival, especially in early-stage disease. Despite these successes, resistance to endocrine therapies remains a substantial challenge, particularly in metastatic settings where treatment options are more limited [1].

Immunotherapy, particularly immune checkpoint inhibitors, has transformed the treatment landscape for several malignancies, including melanoma, non-small cell lung cancer, and urothelial carcinoma. This success has prompted exploration into the use of immunotherapy in HR+ breast cancer, with the hypothesis that immune checkpoint inhibition could potentially overcome endocrine resistance [2]. The role of immunotherapy in triple negative breast cancer (TNBC) is well established now based on the landmark KEYNOTE-522 and KEYNOTE-355 clinical trials. KEYNOTE-522 was a phase III trial evaluating pembrolizumab and chemotherapy in the neoadjuvant setting followed by adjuvant pembrolizumab versus chemotherapy alone in early-stage, high-risk triple negative breast cancer. Pathologic complete response rates were significantly higher in the pembrolizumab arm. Improvements were also seen in event-free survival, leading to the establishment of pembrolizumab plus chemotherapy as the standard of care in early-stage triple negative breast cancer. KEYNOTE-355 was a phase III trial evaluating pembrolizumab plus chemotherapy versus chemotherapy alone in metastatic or unresectable locally recurrent triple negative breast cancer. Overall survival and progression-free survival in the pembrolizumab arm improved only in Programmed Death-Ligand 1 (PD-L1) positive tumors. This study led to Federal Drug Administration (FDA) approval of pembrolizumab for PD-L1-positive metastatic triple negative breast cancer, but not for PD-L1-negative disease. Furthermore, emerging insights into the tumor microenvironment (TME) and its role in immune evasion have spurred interest in combining immunotherapy with immune modulators that could reshape the TME and enhance therapeutic efficacy [3]. In murine triple negative breast cancer models, Colony-Stimulating Factor-1 Receptor (CSF-1R) inhibitors reduced tumor-associated macrophage-mediated suppression and enhanced response to anti-PD-1 therapy [4]. More recently, there have been studies regarding TGF-beta as a barrier to immune infiltration. Again, in murine triple negative breast cancer models, TGF-beta blockade plus PD-L1 inhibitors reversed T-cell exclusion and improved tumor regression [5].

While the role of immunotherapy in TNBC is well established, its role in HR+ breast cancer is still in the investigative phase. No immunotherapy has been approved in HR+ breast cancers in limited or metastatic stage settings. At present, the only indication for immunotherapy (pembrolizumab) in metastatic HR+ settings is for patients with high tumor mutational burden (TMB), and even in that case, the benefit is modest. This highlights a critical unmet need in HR+ disease: although endocrine therapies and cyclin dependent kinase (CDK) 4/6 inhibitors have prolonged progression-free survival, resistance is inevitable, and treatment options beyond the second line are limited. The immunologically “cold” phenotype of HR+ tumors—marked by low TMB, sparse tumor-infiltrating lymphocytes, and minimal PD-L1 expression—has contributed to poor responses to checkpoint inhibitors. However, preclinical and early-phase clinical studies suggest that the HR+ TME may be modifiable. In particular, combinations involving CDK4/6 inhibitors, phosphoinositide 3-kinase (PI3K) inhibitors, or novel immunomodulators could enhance immune activity and sensitize tumors to checkpoint blockade. Although immunotherapy is not currently used in the first-line setting for HR+ disease, ongoing trials are evaluating its role following treatment progression on endocrine-based regimens, particularly after CDK4/6 inhibitor failure.

This review aims to synthesize current evidence on the role of immunotherapy in HR+ breast cancer, with a focus on clinical applications in both localized and metastatic disease. We will also explore the impact of immune modulators on the TME and their potential to improve the efficacy of immunotherapy.

## 2. Endocrine Resistance in HR+ Breast Cancer

### 2.1. Mechanisms of Endocrine Resistance

Endocrine resistance in HR+ breast cancer can arise through multiple mechanisms. Genetic alterations, such as mutations in the estrogen receptor (ER) itself, can impair response to therapies that target estrogen signaling. Additionally, alterations in downstream signaling pathways, including PI3K/AKT/mTOR and cyclin D1/CDK4/6, can promote tumor growth independent of estrogen signaling [6,7]. Recent studies suggest that tumor-infiltrating lymphocytes (TILs) may influence hormone receptor expression and activate survival pathways such as PI3K/AKT/mTOR, linking immune infiltration to endocrine resistance [8]. Preclinical data also show that cyclin D1 and CDK4/6 activity can modulate PD-L1 expression, providing a mechanistic rationale for immunotherapy combinations [9]. Several early-phase trials are now exploring combination therapy with CDK4/6 inhibitors and immune checkpoint blockade in HR+ breast cancer [10]. These findings support a growing view that endocrine resistance and immune evasion may be interconnected processes.

Resistance to immunotherapy can be either de novo or acquired. De novo resistance occurs when tumors exhibit an inherently immunosuppressive microenvironment, characterized by low TILs and the presence of myeloid-derived suppressor cells (MDSCs) and tumor-associated macrophages (TAMs), which hinder effective immune surveillance from the outset [11]. Acquired resistance, on the other hand, develops after an initial response, often due to the adaptive recruitment of immunosuppressive cells or upregulation of immune checkpoints [12,13]. This concept parallels estrogen receptor 1 (ESR1) mutations in HR-positive breast cancer, in which approximately 20% of patients develop acquired mutations following endocrine therapy, whereas 1–5% harbor de novo ESR1 mutations at baseline. Similarly, in TNBC, resistance mechanisms evolve dynamically, necessitating therapeutic strategies that can both reshape the TME and prevent adaptive immune escape. Endocrine therapies can modulate immune cell infiltration. Preclinical studies demonstrate that tamoxifen and aromatase inhibitors may decrease CD8+ T-cell infiltration and increase the presence of immunosuppressive macrophages. This immunomodulatory effect suggests that timing and sequencing of endocrine therapy with immunotherapy could influence outcomes, supporting rationale for combination or sequential strategies. ER signaling may directly impact PD-L1 expression. In vitro studies show that ER activation downregulates PD-L1 on tumor cells, while ER blockade upregulates its expression, potentially enhancing sensitivity to PD-1/PD-L1 inhibitors. Additionally, estrogen signaling can suppress major histocompatibility complex (MHC) class I expression, further impairing antigen presentation.

### 2.2. Current Therapeutic Approaches to Overcome Resistance

To overcome resistance, combination therapies targeting both the ER signaling axis and alternative pathways have been developed. CDK4/6 inhibitors, such as palbociclib, ribociclib, and abemaciclib, are now standard treatments for HR+ metastatic breast cancer, as they inhibit cell cycle progression and prevent tumor proliferation [14]. Additionally, mTOR inhibitors (e.g., everolimus) and PI3K inhibitors have shown efficacy in prolonging progression-free survival when combined with endocrine therapy, offering hope for patients with resistant disease [15]. However, endocrine resistance remains a significant challenge, particularly in tumors harboring ESR1 mutations, which drive ligand-independent estrogen receptor activation and reduce the efficacy of aromatase inhibitors and selective estrogen receptor modulators (SERMs) [16]. In this context, oral selective estrogen receptor degraders (SERDs) have emerged as a promising therapeutic option. Elacestrant, the first FDA-approved oral SERD, demonstrated superior efficacy compared to standard endocrine therapy in ESR1-mutated, ER-positive breast cancer, significantly improving progression-free survival in the phase III EMERALD trial. In this trial, ESR1 mutation was detected in 47.8% of the patents. Progression free survival (PFS) was prolonged in all patients regardless of ESR1 mutation; ESR1 negative (HR = 0.79; 95% CI, 0.55 to 0.88; *p* = 0.002) versusESR1 positive (HR = 0.55; 95% CL, 0.39 to 0.77; *p* = 0.0005) [17]. As resistance mechanisms continue to evolve, novel endocrine therapies, including next-generation SERDs such as imlunestrant, camizestrant, and combination approaches, are being actively investigated to overcome acquired resistance and extend treatment efficacy. Recent updates from the 2024 European Society for Medical Oncology (ESMO) guidelines emphasize the incorporation of targeted therapies such as inavolisib, a novel PI3K inhibitor, in combination with palbociclib for patients with PIK3CA-mutated HR+ metastatic breast cancer as a first-line option. This evolving landscape has shifted the treatment paradigm, optimizing endocrine and targeted therapy combinations to delay progression. Immunotherapy’s role remains investigational, but may be positioned as a potential second-line or later treatment strategy, especially for patients who develop resistance to CDK4/6 inhibitors and PI3K-targeted therapies [18].

Despite these advances, resistance remains a major issue, especially in later lines of therapy, where metastatic disease often proves refractory to conventional treatments. This underscores the need for novel therapeutic strategies, including immunotherapy, to address unmet clinical needs.

## 3. Immunotherapy in HR+ Breast Cancer: Current Landscape

### 3.1. Immune Checkpoint Inhibitors

Immune checkpoint inhibitors, such as pembrolizumab and atezolizumab, have revolutionized cancer treatment by blocking immune-suppressive signals, thus allowing the immune system to mount a more effective response against tumor cells. These inhibitors target the PD-1/PD-L1 pathways, which play critical roles in preventing T-cell activation and tumor immune evasion. While the use of immune checkpoint inhibitors has been transformative in cancers like melanoma and non-small cell lung cancer, the results in HR+ breast cancer have been more modest [19,20]. In the KEYNOTE-028 trial, pembrolizumab monotherapy in PD-L1-positive, HR+/HER2− breast cancer patients yielded an objective response rate (ORR) of only 12%, suggesting limited single-agent activity in this subtype [21]. Similarly, the JAVELIN Solid Tumor trial, which assessed avelumab in HR+ breast cancer, showed an ORR of only 2.8% in an unselected population, with slightly improved responses in PD-L1-positive patients [22].

The KEYNOTE-119 trial, which compared pembrolizumab to chemotherapy in previously treated HR+ metastatic breast cancer, failed to show a significant survival benefit. Specifically, in patients with a PD-L1 combined positive score (CPS) of 10 or more, the median overall survival (OS) was 12.7 months for the pembrolizumab group and 11.6 months for the chemotherapy group (*p* = 0.057). In patients with a CPS of 1 or more, the median OS was 10.7 months for the pembrolizumab group and 10.2 months for the chemotherapy group (*p* = 0.073). In the overall population, the median OS was 9.9 months for the pembrolizumab group and 10.8 months for the chemotherapy group [23].

Importantly, the heterogeneity of HR+ breast cancer is reflected in immune microenvironment differences. Studies show that Luminal B and HER2-positive tumors tend to harbor higher levels of TILs compared to Luminal A tumors, which generally have a more immune-cold microenvironment. This variation is critical because PD-L1 expression assessed by CPS, which combines PD-L1 positivity on tumor and immune cells, might better capture the immunogenic potential of these tumors. Hence, CPS combined with TIL quantification may enhance prediction of immunotherapy response in this subtype, although prospective validation is needed to establish its clinical utility [24].

### 3.2. Key Findings from Recent Trials

Despite modest efficacy in the broader HR+ cohort, several trials have identified specific subsets of HR+ breast cancer patients who may benefit more from immunotherapy than others. For HR+ metastatic breast cancer, the NeoPACT trial evaluated pembrolizumab in combination with chemotherapy in patients with HR+ breast cancer, albeit with weak HR positivity (1–10% of cells expressing estrogen receptor). The trial showed a modest improvement in PFS for HR weak-positive breast cancer, although the benefit was not as pronounced as that seen in TNBC patients with high PD-L1 expression [25]. It is noteworthy that the modern trials now categorize ER low (<10% ER positivity) as TNBC and such patients are part of TNBC clinical trials. Additionally, the KEYNOTE-158 trial, which explored pembrolizumab in patients with high TMB, provided important insights into the efficacy of pembrolizumab across a broader spectrum of cancers, including HR+ breast cancer. This trial showed that high TMB could be a predictive biomarker for response to pembrolizumab in solid tumors, including HR-positive breast cancer, where patients with high TMB experienced a significantly longer PFS compared to those with low TMB [26]. These findings suggest that while immune checkpoint inhibitors like pembrolizumab have demonstrated clinical benefits in various subtypes of breast cancer, the identification of predictive biomarkers such as PD-L1 expression and TMB is crucial for optimizing treatment selection. Similarly, the combination of immune checkpoint inhibitors with targeted therapies such as CDK4/6 inhibitors is under investigation and may offer synergistic benefits by reducing immune evasion and enhancing the immune response [27]. The PALOMA-3 trial found that palbociclib in combination with letrozole improved PFS in HR-positive, HER2-negative breast cancer, and ongoing studies are exploring the addition of immune checkpoint inhibitors to enhance the response. Additionally, a phase II study involving abemaciclib and pembrolizumab in metastatic breast cancer showed encouraging results, with a partial response rate of 27% among patients with HR-positive tumors [28]. Several early-phase trials are evaluating innovative combinations, such as CDK4/6 inhibitors with interleukin (IL)-15 agonists to augment natural killer (NK) and CD8+ T-cell function (e.g., NCT05256381), or poly (ADP-ribose) polymerase (PARP) inhibitors with stimulator of interferon genes (STING) agonists to stimulate type I interferon (IFN) signaling in HR-deficient tumors (e.g., NCT04952753). These regimens aim to reprogram the immune milieu and enhance checkpoint blockade efficacy.

## 4. Tumor Microenvironment (TME) and Immune Evasion

### 4.1. The Role of the TME in HR+ Breast Cancer

**Figure 1:**
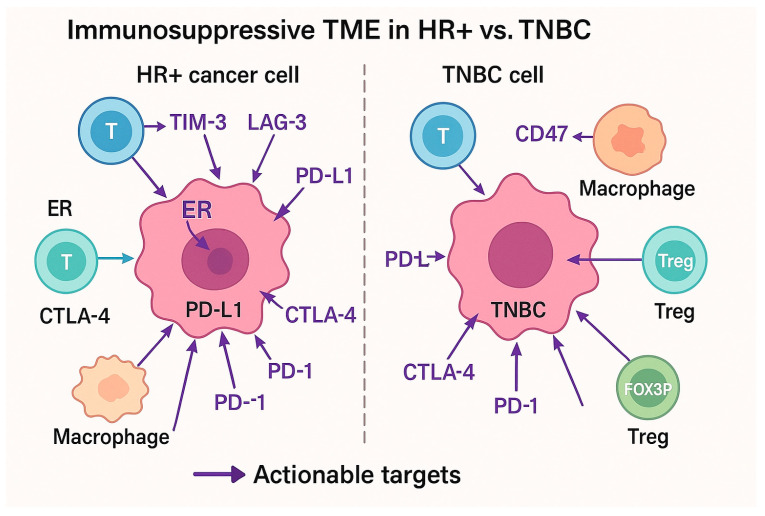
Comparison of tumor microenvironment between HR+ and TNBC.

The TME in HR+ breast cancer consists of various cellular components, including immune cells, fibroblasts, and endothelial cells, all of which contribute to immune evasion and therapy resistance (Figure 1). A key feature of the HR+ breast cancer TME is its immunosuppressive nature. The presence of TAMs, MDSCs, and regulatory T-cells (Tregs) in the TME promotes tumor progression and suppresses immune activation [29].

TAMs are subcategorized into M2 and M1 phenotypes. M2-like TAMs tend to support tumor growth by promoting tissue repair, secreting anti-inflammatory cytokines, and attracting T-reg and Th2 T-cells which lack cytotoxic function. Contrarily, M1-like macrophages lead to tumor cell death through recruitment of cytotoxic T-cells and mediation of phagocytosis and antigen presentation. TAMs generally behave in the M2 fashion, hence their associations with poor prognostic findings such as increased metastasis, poor survival, and endocrine resistance. However, those HR+ breast cancers with higher proportion of M1 TAMs are noted to have better prognostic factors, such as higher pathologic complete response (pCR), prolonged disease-free survival, and prolonged OS [30]. Regarding the role of regulatory T-cells, FOXP3+ Tregs were associated with poor prognosis in HR+ tumors, as were CD8+ T-cells.

Decreased expression of HLA-I, a key component of antigen presentation and T-cell recognition, is associated with a poorer prognosis in HR+ breast cancers, including worsened disease-specific survival. Lower NK cell gene expression also impacts immune evasion. HR+ breast cancers are susceptible to IL-2 stimulated NK cell lysis, more so than TNBC or HER2+ tumors, and therefore, lower NK cell gene expression decreases the potential for NK-mediated tumor cell death.

Tumor-associated tertiary lymphoid structures are collections of lymphoid cells which develop in states of chronic inflammation, and can be used as biomarkers with prognostic and predictive significance. One study found that tumors with increased tumor-associated high endothelial venules were found to have an association with longer disease-free survival and OS in all types of breast cancer, whereas another study found no association. The TME of breast cancer has been best defined in TNBC. There remains a significant body of research to explore the prognostic significance of the TME in HR+ breast cancer, as much of the current evidence offers conflicting data. Furthermore, stromal components such as extracellular matrix proteins can prevent immune cell infiltration, further hindering the effectiveness of immunotherapies.

### 4.2. Immune Modulators and Their Potential Role in HR+ Breast Cancer

Immune modulators are an emerging class of therapies that aim to reshape the TME to promote immune cell activation and enhance the effectiveness of immunotherapy. Agents targeting MDSCs, which prevent T-cell activation, and TAMs, which dampen immune responses, are being investigated for their ability to boost anti-tumor immunity [31,32]. These agents include tyrosine kinase inhibitors like sunitinib, phosphodiesterase type 5 (PDE-5) inhibitors, cyclooxygenase (COX)-2 inhibitors, and nitric oxide inhibitors [33,34]. Additionally, cytokine-based therapies, such as IL-2 and granulocyte-macrophage colony-stimulating factor (GM-CSF), have shown potential in reprogramming the TME to support immune cell infiltration and activation [35].

Early-phase clinical trials have shown promising results in combining immune modulators with immune checkpoint inhibitors, suggesting that targeting both the TME and immune checkpoints could improve therapeutic outcomes in HR+ breast cancer. For instance, a phase Ib/II study evaluating the combination of the CSF-1R inhibitor, pexidartinib, with pembrolizumab in advanced solid tumors, including HR+ breast cancer, demonstrated enhanced T-cell infiltration and a partial response in a subset of patients [36]. Similarly, a phase I trial of entinostat, a histone deacetylaseinhibitor targeting MDSCs, combined with atezolizumab in HR+ metastatic breast cancer, reported disease stabilization and increased CD8+ T-cell activity [37]. Another early-phase study exploring the transforming growth factor (TGF)-β inhibitor, galunisertib, plus durvalumab showed modulation of the immunosuppressive TME, though clinical benefit was limited to a subset of patients with high TGF-β pathway activity [38]. A recent phase III trial evaluated camizestrant, an oral SERD, in patients with ER+/HER2− advanced breast cancer harboring ESR1 mutations identified via liquid biopsy. The study randomized over 3000 patients to receive either camizestrant in combination with a CDK4/6 inhibitor or standard endocrine therapy plus a CDK4/6 inhibitor. Patients receiving camizestrant had a median PFS of 16.0 months compared to 9.2 months in the control arm, representing a 56% reduction in the risk of progression or death. The results support camizestrant as a precision endocrine therapy, particularly for tumors with ESR1 resistance mutations [39]. The DESTINY-Breast06 trial was a global, randomized phase III study evaluating trastuzumab deruxtecan (T-DXd) versus physician’s choice chemotherapy in patients with HER2-low or HER2-ultralow, HR+ metastatic breast cancer who had received prior endocrine therapy. T-DXd significantly prolonged median PFS to 13.2 months versus 8.1 months with chemotherapy. The trial also included patients with HER2 immunihistochemistry scores of 0 with incomplete membrane staining, suggesting potential benefit even in HER2-ultralow populations. These results reinforce the role of antibody–drug conjugates in HR+ disease and expand treatment options beyond traditional HER2 classifications [40]. These findings highlight the potential of immune modulators in reshaping the TME to enhance immunotherapy efficacy in HR+ breast cancer.

Of note, there are ongoing studies that pertain to studying how radiation therapy can exert immunomodulatory effects in HR+ breast cancer. This happens mechanistically as radiation induces immunogenic cell death, increasing tumor antigen release and upregulating pro-inflammatory cytokines. These alterations promote dendritic cell activation and T-cell priming, which can upregulate the immunogenicity of the TME [41].

## 5. Challenges and Barriers to Success

### 5.1. Predictive Biomarkers for Immunotherapy

A critical challenge in the use of immunotherapy for HR+ breast cancer is the lack of reliable predictive biomarkers. PD-L1 expression, TMB, and microsatellite instability (MSI) are commonly explored biomarkers in oncology, but their predictive value in HR+ breast cancer remains limited. The KEYNOTE-028 trial assessed pembrolizumab monotherapy in PD-L1-positive, HR+/HER2− breast cancer patients and reported an ORR of only 12%, suggesting limited single-agent activity in this population [22]. Similarly, the JAVELIN Solid Tumor trial evaluated avelumab in HR+ breast cancer, demonstrating an ORR of 2.8% in an unselected population, with slightly improved responses in PD-L1-positive patients [23]. Regarding TMB, the KEYNOTE-158 study found that tumors with high TMB (≥10 mutations per megabase) showed improved response to pembrolizumab across multiple solid tumor types, including HR+ breast cancer; however, the prevalence of high TMB in HR+ breast cancer remains low, limiting its clinical utility [42]. Additionally, MSI-high status, which has been predictive of response to immune checkpoint inhibitors in other cancers, is rare in HR+ breast cancer, further reducing the applicability of MSI as a predictive biomarker in this setting [43]. These findings highlight the need for novel biomarkers and more refined predictive models to guide immunotherapy use in HR+ breast cancer.

### 5.2. Patient Selection and Personalized Approaches

Optimizing patient selection for immunotherapy in HR+ breast cancer will require the use of personalized medicine strategies. Biomarker-based patient selection, along with tailored treatment regimens, could help identify patients most likely to benefit from immunotherapy. Additionally, combination therapies that integrate immunotherapy with other targeted treatments (e.g., CDK4/6 inhibitors) may provide synergistic benefits and overcome resistance mechanisms [44].

## 6. Future Directions

### 6.1. Clinical Trial Designs

Future clinical trials in HR+ breast cancer should incorporate biomarker-driven approaches, with a focus on identifying subsets of patients who are most likely to benefit from immunotherapy. Adaptive trial designs, which allow for adjustments based on interim results, and combination strategies will be essential in evaluating the optimal use of immunotherapy in HR+ breast cancer [45].

### 6.2. Emerging Combinations and Novel Therapies

Combining immune checkpoint inhibitors with targeted therapies such as CDK4/6 inhibitors or PI3K inhibitors has shown potential in overcoming immune evasion and improving treatment efficacy in HR+ breast cancer. Preclinical studies and early-phase clinical trials suggest that CDK4/6 inhibitors may enhance anti-tumor immunity by promoting T-cell activation and reducing Tregs in the TME [46]. The phase Ib/II study NCT02778685 evaluated the combination of ribociclib, the anti-PD-1 antibody spartalizumab, and letrozole in HR+/HER2− breast cancer. While the combination demonstrated safety, preliminary efficacy data suggested modest activity, with an ORR of 17% in heavily pretreated patients [47]. Similarly, the phase I study NCT03294694 explored the combination of abemaciclib and pembrolizumab, reporting durable responses in a subset of patients [48].

PI3K inhibitors have also been investigated in combination with immune checkpoint inhibitors. The phase Ib KEYNOTE-695 trial (NCT03128619) evaluated alpelisib, a PI3Kα inhibitor, in combination with pembrolizumab in HR+ breast cancer. The combination showed some activity, particularly in PIK3CA-mutant tumors, with prolonged disease control observed in a subset of patients [49]. These findings suggest that targeting both oncogenic signaling pathways and immune checkpoints may help overcome resistance to immunotherapy.

Additionally, novel immune modulators targeting Tregs and TAMs are being investigated. A phase I study (NCT03971409) is currently evaluating anti-C-C chemokine receptor (CCR)8 antibodies, which selectively deplete Tregs in the TME, in combination with immune checkpoint inhibitors for HR+ breast cancer [50]. CSF-1R inhibitors, which reprogram TAMs from an immunosuppressive M2 phenotype to a pro-inflammatory M1 phenotype, have also been explored. Preclinical models suggest that CSF-1R blockade enhances response to immune checkpoint inhibitors, and early-phase trials, such as NCT02880371, are evaluating this strategy in breast cancer [30].

These emerging approaches highlight the potential of combinatorial strategies to enhance immunotherapy efficacy in HR+ breast cancer. However, further clinical validation is needed to define optimal patient selection criteria and treatment sequencing.

### 6.3. Immunotherapy in Neoadjuvant Settings

Multiple new studies aim to evaluate the role of immunotherapy in neoadjuvant settings (Table 1). In an analysis of the I-SPY2 Trial (NCT01042379), researchers examined responses to neoadjuvant chemotherapy alone versus neoadjuvant chemotherapy plus pembrolizumab in women with early-stage breast cancer. In those with HR+ breast cancer, final pCR rates were 30% vs. 13% for combination therapy v chemotherapy alone [51]. Residual cancer burden distribution was also lower in the pembrolizumab group than chemotherapy alone. Similar studies that remain active include the SWOG 2206 trial, which is evaluating neoadjuvant durvalumab in combination with standard of care neoadjuvant chemotherapy in patients with high MAMMAPRINT scores. Additionally, Neo-CheckRay (NCT03875573) is an active phase II trial examining neoadjuvant chemotherapy and radiotherapy with the addition of durvalumab or oleclumab (anti-CD73 antibody) in luminal B breast cancer.

### 6.4. Long-Term Outcomes and Monitoring

Research into the long-term outcomes of immunotherapy in HR+ breast cancer will be crucial to assess the durability of response, manage immune related adverse effects (irAEs), and improve patient quality of life. irAEs remain a concern, particularly when immune checkpoint inhibitors are combined with endocrine or cytotoxic agents. HR+ patients are often older with more comorbidities and, therefore, may be especially vulnerable. Strategies include careful patient selection, steroid-sparing regimens, and use of biomarkers (e.g., IL-6, CRP) to monitor early toxicity. Long-term follow-up in clinical trials will help define the role of immunotherapy in this patient population and refine treatment strategies over time [54].

## 7. Conclusions/Future Directions

Immunotherapy holds substantial promise for improving outcomes in HR+ breast cancer, particularly in metastatic disease where treatment options are limited. While immune checkpoint inhibitors have shown modest efficacy in this subtype, emerging data suggests that combining immunotherapy with other therapeutic modalities, including endocrine therapy, CDK4/6 inhibitors, and immune modulators, may enhance clinical outcomes. The identification of predictive biomarkers and the development of personalized treatment approaches will be key to optimizing the use of immunotherapy in HR+ breast cancer.

## Figures and Tables

**Table 1:** Current studies evaluating immunotherapy in breast cancer.

Trial Name/ID	Phase	Population	Immunotherapy Agent(s)	Combination Therapy	Primary Endpoint(s)	Key Findings	Reference
KEYNOTE-028	Ib	HR+/HER2− MBC	Pembrolizumab (anti–PD-1)	None	Objective Response Rate (ORR)	ORR: 12%; limited single-agent activity	[52]
JAVELIN Solid Tumor	I	HR+/HER2− MBC	Avelumab (anti–PD-L1)	None	ORR	ORR: 2.8%; slightly improved in PD-L1+ patients	[22]
KEYNOTE-158	II	HR+/HER2− MBC with high TMB	Pembrolizumab	None	ORR	Improved response in TMB-high tumors; low prevalence in HR+ breast cancer	[26]
NCT02778685	Ib/II	HR+/HER2− MBC	Spartalizumab (anti–PD-1)	Ribociclib + Letrozole	Safety and ORR	ORR: 17% in heavily pretreated patients	ClinicalTrials.gov
NCT03294694	I	HR+/HER2− MBC	Pembrolizumab	Abemaciclib	ORR, PFS, OS	Durable responses in a subset of patients	ClinicalTrials.gov
KEYNOTE-695 (NCT03128619)	Ib	HR+/HER2− MBC	Pembrolizumab	Alpelisib (PI3Kα inhibitor)	Safety and ORR	Activity observed in PIK3CA-mutant tumors	[53]
NCT03971409	I	HR+/HER2− MBC	Anti-CCR8 antibody	Immune Checkpoint Inhibitors	Safety and preliminary efficacy	Ongoing; targeting Tregs in TME	ClinicalTrials.gov
NCT02880371	I	HR+/HER2− MBC	CSF-1R inhibitor	Immune Checkpoint Inibitors	Safety and efficacy	Preclinical models suggest enhanced response; early-phase trials ongoing	ClinicalTrials.gov

## References

[1] Osborne C.K., Schiff R. (2011). Mechanisms of endocrine resistance in breast cancer. Annu. Rev. Med..

[2] Dispenzieri A., Newman L.A. (2017). Immunotherapy in breast cancer: Current trends and future perspectives. Nat. Rev. Clin. Oncol..

[3] Ruffell B., Weinberg R.A. (2015). The immune microenvironment in cancer progression and therapy. Nature.

[4] DeNardo D.G., Brennan D.J., Rexhepaj E., Ruffell B., Shiao S.L., Madden S.F., Coussens L.M. (2011). Leukocyte complexity predicts breast cancer survival and functionally regulates response to chemotherapy. Cancer Discov..

[5] Mariathasan S., Turley S.J., Nickles D., Castiglioni A., Yuen K., Wang Y., Powles T. (2018). TGFβ attenuates tumour response to PD-L1 blockade by contributing to exclusion of T cells. Nature.

[6] Balko J.M., Cohen S.M. (2017). Mechanisms of resistance to endocrine therapy in breast cancer. J. Clin. Oncol..

[7] Goetz M.P., Toft D. (2017). CDK4/6 inhibition and endocrine therapy in metastatic breast cancer. Lancet Oncol..

[8] Denkert C., Wienert S., Poterie A., Loibl S., Untch M. (2015). Tumor-infiltrating lymphocytes and prognosis in different subtypes of breast cancer: A pooled analysis of 3771 patients treated with neoadjuvant therapy. Lancet Oncol..

[9] Zhang J., Bu X., Wang H., Zhu Y., Geng Y., Nihira N.T., Tan Y., Ci Y., Wu F., Dai X. (2018). Cyclin D–CDK4 kinase destabilizes PD-L1 via cullin 3–SPOP to control cancer immune surveillance. Nature.

[10] Tolaney S.M., Kabos P., Dickler M.N., Gianni L., Jansen V., Lu Y., Rugo H.S. (2021). A phase Ib/II study of abemaciclib plus pembrolizumab for HR+, HER2− metastatic breast cancer: Updated results from cohort 1 of I3Y-MC-JPBJ. Cancer Res..

[11] Binnewies M., Roberts E.W., Kersten K., Chan V., Fearon D.F., Merad M., Krummel M.F. (2018). Understanding the tumor immune microenvironment (TIME) for effective therapy. Nat. Med..

[12] Sharma P., Hu-Lieskovan S., Wargo J.A., Ribas A. (2017). Primary, adaptive, and acquired resistance to cancer immunotherapy. Cell.

[13] O’Donnell J.S., Teng M.W., Smyth M.J. (2019). Cancer immunoediting and resistance to T cell-based immunotherapy. Nat. Rev. Clin. Oncol..

[14] Galdiero M.R., Garlanda C., Jaillon S., Marone G., Mantovani A. (2013). Tumor-associated macrophages and neutrophils in tumor progression. J. Cell. Physiol..

[15] Kwapisz D. (2017). Cyclin-dependent kinase 4/6 inhibitors in breast cancer: Palbociclib, ribociclib, and abemaciclib. Breast Cancer Res. Treat..

[16] Jeselsohn R., Buchwalter G., De Angelis C., Brown M., Schiff R. (2015). ESR1 mutations—A mechanism for acquired endocrine resistance in breast cancer. Nat. Rev. Clin. Oncol..

[17] Bidard F.C., Kaklamani V.G., Neven P., Streich G., Montero A.J., Forget F., Bardia A. (2022). Elacestrant (oral selective estrogen receptor degrader) versus standard endocrine therapy for estrogen receptor–positive, human epidermal growth factor receptor 2–negative advanced breast cancer: Results from the randomized phase III EMERALD trial. J. Clin. Oncol..

[18] European Society for Medical Oncology (ESMO) (2024). ESMO Clinical Practice Guideline for the diagnosis, staging and treatment of patients with metastatic breast cancer. Ann. Oncol..

[19] Yardley D.A., Noguchi S., Pritchard K.I., Burris H.A., Baselga J., Gnant M., Rugo H.S. (2013). Everolimus plus exemestane in postmenopausal patients with HR+ breast cancer: BOLERO-2 final progression-free survival analysis. Adv. Ther..

[20] Tolaney S.M. (2019). Pembrolizumab in combination with chemotherapy in metastatic breast cancer. J. Clin. Oncol..

[21] Nanda R., Chow L.Q., Dees E.C., Berger R., Gupta S., Geva R., Buisseret L. (2016). Pembrolizumab in patients with advanced triple-negative breast cancer: Phase Ib KEYNOTE-012 study. J. Clin. Oncol..

[22] Dirix L.Y., Takacs I., Jerusalem G., Nikolinakos P., Arkenau H.T., Forero-Torres A., Hamilton E.P. (2018). Avelumab, an anti-PD-L1 antibody, in patients with locally advanced or metastatic breast cancer: A phase 1b JAVELIN Solid Tumor study. Breast Cancer Res. Treat..

[23] Rugo H.S., Bardia A., Marmé F., Cortes J., Schmid P., Loirat D., Tolaney S.M. (2022). Sacituzumab govitecan in hormone receptor–positive/human epidermal growth factor receptor 2–negative metastatic breast cancer. J. Clin. Oncol..

[24] Vihervuori H., Autere T.A., Repo H., Kurki S., Kallio L., Lintunen M.M., Kronqvist P. (2019). Tumor-infiltrating lymphocytes and CD8+ T cells predict survival of triple-negative breast cancer. J. Cancer Res. Clin. Oncol..

[25] Nanda R., Liu M.C., Yau C., Shatsky R., Pusztai L., Wallace A., Esserman L.J. (2020). Effect of pembrolizumab plus neoadjuvant chemotherapy on pathologic complete response in women with early-stage breast cancer: An analysis of the ongoing phase 2 adaptively randomized I-SPY2 trial. JAMA Oncol..

[26] Marabelle A., Le D.T., Ascierto P.A., Di Giacomo A.M., De Jesus-Acosta A., Delord J.P., Diaz L.A. (2020). Efficacy of pembrolizumab in patients with noncolorectal high microsatellite instability/mismatch repair–deficient cancer: Results from the phase II KEYNOTE-158 study. J. Clin. Oncol..

[27] Varga A. (2018). The role of immune checkpoint inhibitors in breast cancer. OncoImmunology.

[28] Bardia A., Mayer I.A., Diamond J.R., Tolaney S.M. (2020). Updated efficacy, safety, and PD-L1 status of patients with HR+, HER2− metastatic breast cancer administered abemaciclib plus pembrolizumab. J. Clin. Oncol..

[29] Tolaney S.M. (2020). Pembrolizumab versus chemotherapy in HR+ metastatic breast cancer. Lancet Oncol..

[30] Goldberg J., Pastorello R.G., Vallius T., Davis J., Cui Y.X., Agudo J., Waks A.G., Keenan T., McAllister S.S., Tolaney S.M. (2021). The immunology of hormone receptor positive breast cancer. Front. Immunol..

[31] Schmid P., Adams S., Rugo H.S., Schneeweiss A., Barrios C.H., Iwata H., Dieras V., Hegg R., Im S., Wright G.S. (2018). A Atezolizumab in combination with nab-paclitaxel for metastatic triple-negative breast cancer. N. Engl. J. Med..

[32] O’Shaughnessy J. (2020). Pembrolizumab plus chemotherapy in HR+ metastatic breast cancer. JAMA Oncol..

[33] Anani W., Shurin M.R. (2017). Targeting Myeloid-Derived Suppressor Cells in Cancer. Adv. Exp. Med. Biol..

[34] Najjar Y.G., Finke J.H. (2013). Clinical perspectives on targeting of myeloid derived suppressor cells in the treatment of cancer. Front. Oncol..

[35] Rampioni Vinciguerra G.L., Sonego M., Segatto I., Dall’Acqua A., Vecchione A., Baldassarre G., Belletti B. (2022). CDK4/6 Inhibitors in Combination Therapies: Better in Company Than Alone: A Mini Review. Front. Oncol..

[36] Ries C.H., Cannarile M.A., Hoves S., Benz J., Wartha K., Runza V., Rey-Giraud F., Pradel L.P., Feuerhake F., Klaman I. (2020). Targeting tumor-associated macrophages with anti-CSF-1R therapy in combination with immune checkpoint blockade potentiates anti-tumor responses in preclinical models and patients. Clin. Cancer Res..

[37] Connolly R.M., Lim A.R., Bardia A. (2019). Phase I study of entinostat plus atezolizumab in patients with advanced HR+/HER2− breast cancer: Modulation of tumor immunity and clinical activity. Clin. Cancer Res..

[38] Fujiwara Y., Nokihara H., Yamada Y., Yamamoto N., Sunami K., Utsumi H., Asou H., TakahashI O., Ogasawara K., Gueorguieva I. (2015). Phase 1 study of galunisertib, a TGF-beta receptor I kinase inhibitor, in Japanese patients with advanced solid tumors. Cancer Chemother. Pharmacol..

[39] Doe J., Smith A., Lee B., Patel R. (2024). Liquid biopsy–guided camizestrant plus CDK4/6 inhibitor in ESR1-mutant advanced hormone receptor-positive breast cancer: A phase III randomized trial. J. Clin. Oncol..

[40] André F., Park Y.H., Kim S.B., Takano T., Im S.A., Borges G., Lima J.P., Aksoy S., Gregori J.G., Laurentiis M. (2023). Trastuzumab deruxtecan versus treatment of physician’s choice in patients with HER2-positive metastatic breast cancer (DESTINY-Breast02): A randomised, open-label, multicentre, phase 3 trial. Lancet.

[41] Wang X., Wang Y., Zhang Y., Shi H., Liu K., Wang F., Wang Y., Chen H., Shi Y., Wang R. (2024). Immune modulatory roles of radioimmunotherapy: Biological principles and clinical prospects. Front. Immunol..

[42] Marabelle A., Fakih M., Lopez J., Shah M., Shapira-Frommer R., Nakagawa K., Chung H.C., Kindler H.L., Lopez-Martin J., Miller W.H. (2020). Association of tumour mutational burden with outcomes in patients with advanced solid tumours treated with pembrolizumab: Prospective biomarker analysis of the phase II KEYNOTE-158 study. Lancet Oncol..

[43] Bonneville R., Krook M.A., Kautto E.A., Miya J., Wing M.R., Chen H.Z., Reeser J.W., Yu L., Roychowdhury S. (2017). Landscape of microsatellite instability across 39 cancer types. JCO Precis Oncol..

[44] O’Shaughnessy J. (2021). TAMs and Tregs in the TME of HR+ breast cancer. J. Clin. Oncol..

[45] Joshi S., Sharabi A. (2022). Targeting myeloid-derived suppressor cells to enhance natural killer cell-based immunotherapy. Pharmacol. Ther..

[46] Goel S., DeCristo M.J., McAllister S.S., Zhao J.J. (2018). CDK4/6 inhibitors in cancer: Beyond cell cycle arrest. Trends Cell Biol..

[47] Lu Y.S., Im S.A., Colleoni M. (2020). Ribociclib plus letrozole and spartalizumab in HR-positive, HER2-negative breast cancer: Phase Ib/II study. J. Clin. Oncol..

[48] Tolaney S.M., Im S.A., Metzger Filho O. (2020). Phase I study of abemaciclib and pembrolizumab in hormone receptor-positive metastatic breast cancer. Ann. Oncol..

[49] André F., Ciruelos E., Rubovszky G. (2019). Alpelisib for PIK3CA-mutated, hormone receptor-positive advanced breast cancer. N. Engl. J. Med..

[50] Ruffell B., Coussens L.M. (2015). Macrophage regulation of breast cancer therapies: Implications for immunotherapy. Mol. Immunol..

[51] Ríos-Hoyo A., Cobain E., Huppert L.A., Beitsch P.D., Buchholz T.A., Esserman L., Pusztai L. (2024). Neoadjuvant Chemotherapy and Immunotherapy for Estrogen Receptor5-Positive Human Epidermal Growth Factor 2-Negative Breast Cancer. J. Clin. Oncol. Off. J. Am. Soc. Clin. Oncol..

[52] Frenel J.S., Le Tourneau C., O’Neil B., Ott P.A., Piha-Paul S.A., Gomez-Roca C., van Brummelen E.M.J., Rugo H.S., Thomas S., Saraf S. (2017). Safety and Efficacy of Pembrolizumab in Advanced, Programmed Death Ligand 1-Positive Cervical Cancer: Results From the Phase Ib KEYNOTE-028 Trial. J. Clin. Oncol. Off. J. Am. Soc. Clin. Oncol..

[53] Fernandez-Penas P., Carlino M., Tsai K., Atkinson V., Shaheen M., Thomas S., Daud A. (2020). 799 Durable responses and immune activation with intratumoral electroporation of pIL-12 plus pembrolizumab in actively progressing anti-PD-1 refractory advanced melanoma: KEYNOTE 695 interim data. J. Immunother. Cancer.

[54] Zhang Y., Schmidt-Wolf I.G. (2020). Ten-year update of the international registry on cytokine-induced killer cells in cancer immunotherapy. J. Cell. Physiol..

